# Early Aneurysm Occlusion Outcomes After Endovascular Treatment: A Descriptive Single-Center Cohort Evaluation

**DOI:** 10.3390/neurolint18090170

**Published:** 2026-09-11

**Authors:** Yasar Ünsal, Kadir Çetinkaya, Mehmet Özgür Özates, Oktay Gürcan, Oktay Algın, Gıyas Ayberk

**Affiliations:** 1 Neurosurgery Department, Bilkent City Hospital, Ankara 06800, Türkiye; yaasar40@gmail.com; 2 Neurosurgery Department, Hıtıt University Corum Erol Olcok Training and Research Hospital, Corum 19040, Türkiye; 3 Neurosurgery Department, Yıldırım Beyazıt University, Ankara 06690, Türkiye; dr.mehmetozgurozates@gmail.com (M.Ö.Ö.); oktay44@yahoo.com (O.G.); giyas67@hotmail.com (G.A.); 4 National MR Research Center, Bilkent University, Ankara 06800, Türkiye; oktay.algin@umram.bilkent.edu.tr; 5 Radiology Department, Medical Faculty of Ankara University, Ankara 06230, Türkiye; 6 Interventional MR Clinical Research and Development Institute, Ankara University, Ankara 06100, Türkiye

**Keywords:** intracranial aneurysm, endovascular treatment, Woven EndoBridge, flow diverter, coil embolization, recurrence, residual aneurysm

## Abstract

Background: Early residual or recurrent aneurysm filling remains an important limitation after endovascular treatment of intracranial aneurysms, particularly in large and complex lesions. This study descriptively evaluated 6-month early residual or recurrent filling after coil embolization, stent-assisted coiling, flow-diverting stents, and the Woven EndoBridge (WEB) device in a single-center cohort. Methods: We retrospectively analyzed 151 patients with 161 intracranial aneurysms treated between January 2019 and July 2023. Follow-up imaging at approximately 6 months was performed using computed tomography angiography or magnetic resonance angiography, with digital subtraction angiography when residual filling was suspected. Aneurysm occlusion was evaluated using the Raymond–Roy Occlusion Classification. Clinical, morphological, anatomical, and treatment-related variables associated with early residual or recurrent aneurysm filling were analyzed. Results: Complete occlusion was achieved in 149 aneurysms (92.5%), whereas early residual or recurrent filling occurred in 12 aneurysms (7.5%). Residual or recurrent filling was observed in 4/35 aneurysms treated with coil embolization (11.4%), 1/58 treated with flow-diverting stents (1.7%), 7/60 treated with the WEB device (11.7%), and none of the 8 aneurysms treated with stent-assisted coiling; overall rates did not differ significantly among treatment modalities (*p* = 0.104). Aneurysm height > 10 mm was significantly associated with early residual or recurrent filling (*p* = 0.005), and mean aneurysm height was greater in aneurysms with residual or recurrent filling than in completely occluded aneurysms (*p* = 0.048). In the ICA subgroup, residual or recurrent filling was more frequent after WEB treatment than after other endovascular modalities (*p* = 0.019). This subgroup signal was based on sparse observations. Conclusions: Aneurysm height > 10 mm was significantly associated with early residual or recurrent aneurysm filling. No statistically significant difference in early residual or recurrent filling was detected among treatment groups. Given the retrospective design, non-random treatment allocation, heterogeneous aneurysm phenotypes, and non-uniform imaging follow-up, these data should be interpreted as a descriptive institutional experience rather than evidence of comparative superiority or equivalence. The ICA subgroup finding is preliminary and hypothesis-generating.

## 1. Introduction

Endovascular treatment (EVT) of intracranial aneurysms using detachable platinum coils was first introduced in the early 1990s with the development of electrically detachable coils by Guglielmi et al. [1]. Although coil embolization remains an established treatment option, the management of large, giant, fusiform, blister-type, and wide-neck aneurysms can be challenging because of coil instability, incomplete neck coverage, coil compaction, and the risk of coil protrusion into the parent artery. To address the limitations of conventional coiling in complex aneurysms, adjunctive endovascular strategies and flow-diversion technologies have been developed [2]. Together with the expanding role of endovascular therapy in aneurysm management, newer technologies such as flow-diverting stents and intrasaccular flow disruption devices have broadened the treatment options for complex intracranial aneurysms [3,4].

The durability of coil embolization is influenced by several factors, including aneurysm morphology, parent vessel anatomy, coil type, packing density, and operator experience. Coil embolization has the advantage of providing immediate aneurysm protection, which is particularly important in ruptured aneurysms. Nevertheless, incomplete occlusion, coil compaction, and persistent inflow at the aneurysm neck may contribute to residual or recurrent aneurysm filling during follow-up.

Stent architecture, including open-cell, closed-cell, braided, double-layer, and micromesh configurations, may influence conformability, wall apposition, pore characteristics, and flow diversion in complex vascular anatomy. Contemporary clinical data on double-layer MicroNet stents further underscore the importance of device design when treating anatomically challenging lesions [5]. Flow-diverting stents are designed to redirect blood flow along the parent artery, promote intra-aneurysmal thrombosis, and support endothelial remodeling across the aneurysm neck. They are particularly useful for sidewall aneurysms, whereas their use in bifurcation aneurysms requires careful consideration because of the need to preserve branch vessels [6]. Unlike coil embolization, aneurysm occlusion after flow diversion is usually progressive and may occur over several months [7]. Flow-diverting devices have been compared with coil embolization in terms of angiographic and clinical outcomes, and their use requires careful patient and aneurysm selection [8]. The requirement for dual antiplatelet therapy before and after stent placement remains an important clinical consideration, particularly in ruptured aneurysms or in patients at increased hemorrhagic risk [9].

Approximately 26–36% of intracranial aneurysms are wide-necked, making treatment technically demanding because of the need to achieve durable aneurysm occlusion while preserving adjacent branch vessels [10]. The WEB device is an intrasaccular flow disruptor developed for the treatment of ruptured and unruptured wide-neck aneurysms. It consists of braided nitinol wires over a platinum core and self-expands within the aneurysm sac. Its low-porosity structure reduces inflow into the aneurysm and promotes thrombosis [11]. Because the WEB device is deployed within the aneurysm sac, it can be particularly useful for wide-neck bifurcation aneurysms and may reduce the need for permanent implants across the parent artery. In selected cases, it may also reduce the need for post-procedural dual antiplatelet therapy [12].

Despite these advances, early residual or recurrent aneurysm filling remains a clinically relevant problem after EVT. The risk of incomplete early occlusion may be influenced by aneurysm morphology, anatomical location, and treatment modality. While longer-term follow-up is crucial for assessing definitive occlusion, the early recanalization or residual filling often occurs within the first few months, making the 6-month time point critical for initial assessment and potential re-intervention decisions [13]. The clinical value of a single-center series such as this is not to identify a universal winner among devices, but to provide a transparent real-world description of early angiographic outcomes across the range of modalities used in routine practice. Such descriptive data may help generate clinically testable hypotheses regarding aneurysm size, presentation, location, and device selection, although they cannot replace balanced comparative studies. Therefore, the aim of this study was to describe 6-month early residual or recurrent aneurysm filling after coil embolization, stent-assisted coiling, flow-diverting stent placement, and WEB treatment in a single-center cohort of intracranial aneurysms. We also explored clinical, morphological, anatomical, and treatment-related factors associated with early residual or recurrent filling. Because treatment allocation was determined by aneurysm phenotype, clinical presentation, and anatomy, the study was not designed to establish comparative superiority among modalities.

## 2. Materials and Methods

### 2.1. Study Design and Ethical Approval

This retrospective single-center study included patients who underwent endovascular treatment for intracranial aneurysms between January 2019 and July 2023. Clinical and radiological data were reviewed from institutional medical records and imaging archives. Pre- and post-treatment evaluations were performed using computed tomography angiography (CTA), contrast-enhanced magnetic resonance angiography (CE-MRA), time-of-flight magnetic resonance angiography (TOF-MRA), and two-dimensional reconstruction when needed.

The clinical variables included age, sex, and hypertension status. Morphological and anatomical variables included aneurysm location, aneurysm height, dome diameter, neck width, and neck-to-dome ratio. The primary unit of analysis was the aneurysm.

This study was conducted in accordance with the principles of the Declaration of Helsinki. Ethical approval was obtained from the Ankara Bilkent City Hospital No. 1 Clinical Research Ethics Committee (date: 16 August 2023; decision no.: E1/3896/2023). The requirement for informed consent was waived because of the retrospective design of the study.

### 2.2. Patient Selection

During the study period, 166 patients with 176 intracranial aneurysms underwent endovascular treatment at our institution. Patients were eligible for inclusion if they had an intracranial aneurysm confirmed by TOF-MRA or another vascular imaging modality, underwent endovascular treatment at our center, and completed approximately 6-month imaging follow-up at our institution.

**Figure 1:**
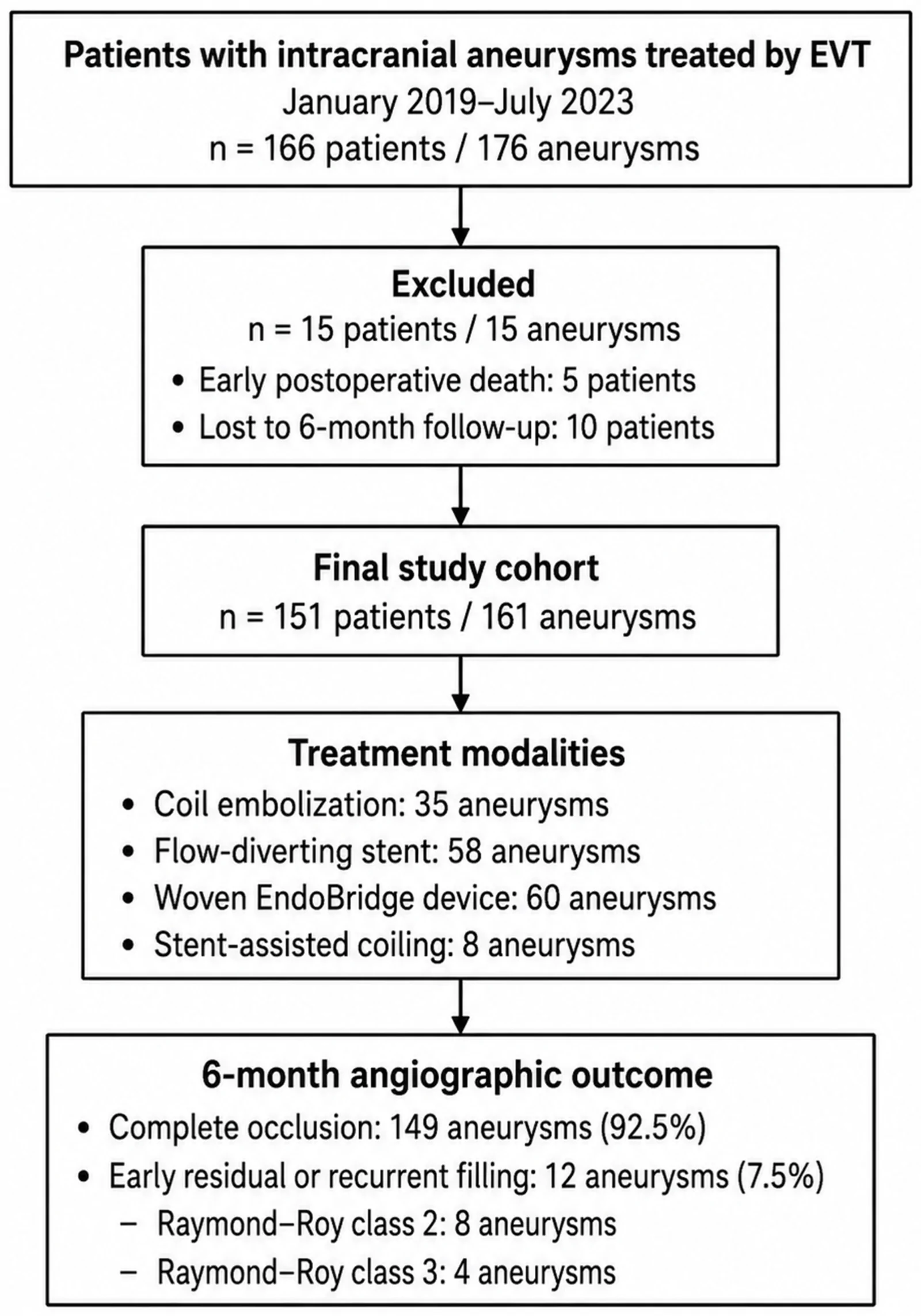
Flowchart of patient and aneurysm selection.

Patients were excluded if the aneurysm had been treated at another center or if baseline or follow-up vascular imaging was unavailable. Fifteen patients were excluded: 5 patients died in the early postoperative period, and 10 patients were lost to 6-month follow-up. The final analysis included 151 patients with 161 aneurysms. Because these five patients died before the scheduled 6-month angiographic follow-up, they were excluded only from the primary angiographic efficacy analysis because a Raymond–Roy Occlusion Classification outcome could not be assigned. All five had presented with ruptured intracranial aneurysms. Three underwent coil embolization, one underwent WEB treatment, and one underwent flow-diverting stent placement. Retrospective medical-record review attributed two deaths to the severity of the presenting subarachnoid hemorrhage and three deaths to complications of underlying medical conditions; none was considered directly related to a technical or intraprocedural complication of endovascular treatment. The patient and aneurysm selection process is summarized in Figure 1.

### 2.3. Endovascular Treatment Strategy

All procedures were performed at a single center by the same neurointerventional team. The choice of endovascular treatment modality was based on aneurysm location, aneurysm morphology, neck configuration, relationship to arterial bifurcations, and parent vessel anatomy. Specifically, coil embolization was generally preferred for small, narrow-necked aneurysms. Stent-assisted coiling was utilized for wide-necked aneurysms where coil stability was a concern. Flow-diverting stents were primarily used for large or giant, fusiform, or dissecting aneurysms, especially in non-bifurcation locations. The WEB device was reserved for wide-necked bifurcation aneurysms, particularly those with complex geometries, following established institutional protocols and neurointerventional team consensus. Treatment modalities included coil embolization, stent-assisted coiling, flow-diverting stent placement, and Woven EndoBridge (WEB) device treatment. For selected wide-necked aneurysms, a single-layer WEB device was used.

### 2.4. Imaging Follow-Up

Follow-up imaging was performed approximately 6 months after endovascular treatment. Because early recanalization or residual aneurysm filling is commonly detected during the first months after endovascular treatment, this time point was selected as the primary angiographic follow-up interval [13,14]. Follow-up assessment was performed using CTA or TOF-MRA. Digital subtraction angiography (DSA) was not performed systematically in all patients; it was obtained selectively when residual aneurysm filling was suspected on CTA or MRA. Consequently, CTA/MRA served as the primary follow-up modalities in this cohort, whereas DSA was used as a problem-solving examination rather than as a uniform reference standard. This ascertainment strategy may have underestimated small neck remnants or subtle recurrent filling and may have inflated apparent complete-occlusion rates, particularly in treatment groups with high observed occlusion rates.

### 2.5. Outcome Definition

Aneurysm occlusion at 6-month follow-up was assessed according to the Raymond–Roy classification [15]. Class 1 was defined as complete aneurysm occlusion with no residual filling, class 2 as residual filling at the aneurysm neck, and class 3 as residual filling within the aneurysm sac. For the purposes of this study, Raymond–Roy class 1 was defined as complete occlusion, whereas Raymond–Roy classes 2 and 3 were defined as early residual or recurrent aneurysm filling. The applicability and validation of the Raymond–Roy classification for non-invasive imaging modalities such as CTA and MRA have been previously established in the literature [16].

### 2.6. Statistical Analysis

Continuous variables were expressed as mean ± standard deviation or median with interquartile range, as appropriate. Categorical variables were expressed as frequencies and percentages. The Student *t*-test or Mann–Whitney U test was used to compare continuous variables between two independent groups, depending on data distribution. One-way analysis of variance or the Kruskal–Wallis test was used for comparisons among more than two groups. When significant differences were identified, appropriate post hoc multiple-comparison tests were applied.

Categorical variables were compared using the chi-square test or Fisher’s exact test, as appropriate. Because only 12 early residual or recurrent filling events occurred, the study had limited statistical power for multivariable modelling and nested subgroup analyses. Subgroup analyses were considered exploratory and hypothesis-generating rather than confirmatory. All statistical analyses were performed using IBM SPSS Statistics for Windows, version 26.0 (IBM Corp., Armonk, NY, USA). A two-sided *p* value of <0.05 was considered statistically significant.

## 3. Results

### 3.1. Patient Flow and Baseline Characteristics

During the study period, 166 patients with 176 intracranial aneurysms underwent endovascular treatment. Fifteen patients were excluded from the analysis: 5 patients died in the early postoperative period, and 10 patients were lost to 6-month follow-up. The five early postoperative deaths were excluded from only the 6-month angiographic efficacy analysis because follow-up imaging was unavailable. All five patients had presented with ruptured intracranial aneurysms; three underwent coil embolization, one underwent WEB treatment, and one underwent flow-diverting stent placement. Two deaths were attributed to the severity of the presenting subarachnoid hemorrhage and three to complications of underlying medical conditions. None were considered directly related to a technical or intraprocedural complication of endovascular treatment. These patients were retained in the overall safety and mortality assessment, yielding an early mortality rate of 5/166 patients (3.0%). The final cohort included 151 patients with 161 aneurysms. The patient and aneurysm selection process is summarized in Figure 1.

During the study period, 166 patients with 176 intracranial aneurysms underwent endovascular treatment. Fifteen patients were excluded because of early postoperative death or loss to 6-month follow-up. The final analysis included 151 patients with 161 aneurysms.

The mean patient age was 57.22 ± 14.07 years, with a median age of 58 years. Eighty-six patients were female (56.95%), and 65 were male (43.04%). Hypertension was present in 83 patients (54.96%) and absent in 68 patients (45.03%). Of the 161 aneurysms included in the final analysis, 20 (12.4%) were ruptured at presentation and 141 (87.6%) were unruptured. The mean aneurysm neck width was 3.04 ± 1.05 mm, the mean neck-to-dome ratio was 0.47 ± 0.21, the mean aneurysm height was 7.97 ± 4.92 mm, and the mean dome diameter was 7.71 ± 5.44 mm.

The distribution of aneurysms by location was as follows: ACoA, 48 aneurysms (29.81%); MCA, 49 aneurysms (30.43%); ICA, 53 aneurysms (32.92%); basilar artery, 7 aneurysms (4.35%); and vertebral artery, 4 aneurysms (2.48%). Treatment modalities included coil embolization in 35 aneurysms (21.74%), flow-diverting stent placement in 58 aneurysms (36.02%), WEB device treatment in 60 aneurysms (37.27%), and stent-assisted coiling in 8 aneurysms (4.97%) (Table 1).

The treatment groups differed intrinsically in their clinical and anatomical indications. Coil embolization was generally used for smaller, narrow-neck aneurysms; flow-diverting stents were preferentially used for large, fusiform, or dissecting lesions; and WEB treatment was reserved mainly for wide-neck bifurcation aneurysms. Accordingly, the treatment groups should not be interpreted as exchangeable cohorts, and the between-modality comparison is descriptive and hypothesis-generating rather than causal.

### 3.2. Six-Month Angiographic Outcomes

At 6-month follow-up, aneurysm occlusion was assessed according to the Raymond–Roy classification. Complete occlusion, defined as Raymond–Roy class 1, was observed in 149 aneurysms (92.55%). Early residual or recurrent aneurysm filling, defined as Raymond–Roy class 2 or 3, was detected in 12 aneurysms (7.45%). Raymond–Roy class 2 residual neck filling was observed in 8 aneurysms (4.97%), whereas Raymond–Roy class 3 residual aneurysm filling was observed in 4 aneurysms (2.48%). Among the aneurysms with class 3 residual filling, retreatment was performed with coil embolization in 1 case, flow-diverting stent placement in 1 case, and WEB treatment in 2 cases.

According to treatment modality, early residual or recurrent filling was detected in 4 of 35 aneurysms treated with coil embolization (11.4%), 1 of 58 aneurysms treated with flow-diverting stents (1.7%), and 7 of 60 aneurysms treated with the WEB device (11.7%). No early residual or recurrent filling was observed among the 8 aneurysms treated with stent-assisted coiling. Overall, early residual or recurrent filling rates did not differ significantly among treatment modalities (*p* = 0.104) (Table 2). This comparison is descriptive because the treatment groups differed substantially in aneurysm size, morphology, location, and clinical indication.

### 3.3. Morphological and Clinical Factors Associated with Early Residual or Recurrent Filling

Aneurysm height was significantly associated with early residual or recurrent filling. Among aneurysms with complete occlusion, 20 of 149 aneurysms (13.4%) had a height > 10 mm, whereas 6 of 12 aneurysms (50.0%) with early residual or recurrent filling had a height > 10 mm (*p* = 0.005). This indicates that aneurysms with a height > 10 mm constituted a significantly larger proportion of those with early residual or recurrent filling (6/12, 50.0%) compared to those with complete occlusion (20/149, 13.4%). When evaluated as a continuous variable, mean aneurysm height was also significantly higher in aneurysms with early residual or recurrent filling than in those with complete occlusion (10.67 ± 5.25 mm vs. 7.75 ± 4.85 mm; *p* = 0.048).

Neck width was not significantly associated with early residual or recurrent filling. Neck width > 4 mm was present in 30 of 149 aneurysms (20.1%) with complete occlusion and in 1 of 12 aneurysms (8.3%) with early residual or recurrent filling (*p* = 0.463). Similarly, no significant differences were observed between aneurysms with and without early residual or recurrent filling in terms of dome diameter, neck-to-dome ratio, or patient age. Sex and hypertension were also not significantly associated with early residual or recurrent filling (Table 3).

### 3.4. Location-Specific Subgroup Analysis

In the location-specific subgroup analysis, the only statistically significant difference was observed in ICA aneurysms. In this subgroup, early residual or recurrent filling was observed in 1 of 2 coil-treated aneurysms, none of 31 flow-diverter-treated aneurysms, 2 of 14 WEB-treated aneurysms, and none of 6 aneurysms treated with stent-assisted coiling (*p* = 0.019). Because of the small number of events and sparse subgroup counts, this finding should be interpreted cautiously and considered hypothesis-generating (Supplementary Table S1).

### 3.5. Procedure-Related Complications

Procedure-related complications are summarized here because they represent clinically relevant outcomes independent of 6-month aneurysm occlusion. No treatment-related mortality was observed among the patients included in the final analysis. No intraprocedural aneurysm rupture, contrast-related nephropathy, or allergic reaction was recorded. Access-site pseudoaneurysm occurred in 12 patients and resolved during follow-up; vasospasm was observed in 36 patients. Six WEB-treated cases developed MCA subsegmental infarcts during the procedure. In one of these cases, MCA branch occlusion due to device malpositioning required thrombectomy and low-profile stent placement.

## 4. Discussion

In this single-center cohort of 151 patients with 161 intracranial aneurysms treated endovascularly, the overall rate of early residual or recurrent aneurysm filling at 6-month follow-up was 7.5%. The corresponding rates were 11.4% after coil embolization, 1.7% after flow-diverting stent placement, 11.7% after WEB treatment, and 0% after stent-assisted coiling. No statistically significant difference in early residual or recurrent filling was detected among treatment modalities. However, because the groups were defined by different anatomical and clinical indications, this finding should be interpreted only as a descriptive comparison within a selected retrospective cohort and not as evidence of equivalent efficacy or comparative superiority. The most consistent finding of the present study was the significant association between aneurysm height > 10 mm and early residual or recurrent filling. In addition, the location-specific subgroup analysis suggested a higher rate of early residual or recurrent filling among ICA aneurysms treated with the WEB device; however, this finding should be interpreted cautiously because of the small number of events.

Residual aneurysm filling after coil embolization is a well-recognized limitation of endovascular therapy. In the present study, early residual or recurrent filling was detected in 4 of 35 aneurysms treated with coil embolization. The durability of coil embolization is influenced by several factors, including coil packing density, aneurysm morphology, neck width, dome size, aneurysm height, and local hemodynamic conditions. Previous studies have reported variable rates of recurrence and reopening after coil embolization, reflecting differences in aneurysm morphology, rupture status, treatment technique, follow-up duration, and imaging protocols [17,18,19]. In line with these reports, aneurysm height was significantly associated with incomplete early occlusion in the present study. Aneurysms with a height > 10 mm had a higher rate of early residual or recurrent filling than smaller aneurysms, and aneurysm height was also significantly greater in aneurysms with incomplete early occlusion when analyzed as a continuous variable.

Flow-diverting stents have expanded the treatment options for complex intracranial aneurysms, particularly sidewall aneurysms. Their mechanism of action depends on hemodynamic flow diversion, progressive intra-aneurysmal thrombosis, and endothelial remodeling across the aneurysm neck [20,21,22]. Although flow diversion can provide durable aneurysm occlusion, branch vessel involvement remains an important consideration, particularly in bifurcation anatomy [23]. Unlike coil embolization, occlusion after flow diversion is usually progressive and may continue to improve beyond the early follow-up period. Previous studies have shown that a proportion of aneurysms remain incompletely occluded at 6 months after flow-diverter treatment, whereas complete occlusion rates may increase substantially during longer follow-up [24,25,26,27,28,29,30]. In the present cohort, only 1 of 58 aneurysms treated with flow-diverting stents showed early residual or recurrent filling, corresponding to a complete occlusion rate of 98.3%. This rate is higher than many previously reported 6-month occlusion rates. This unexpectedly high occlusion rate warrants careful consideration. It is plausible that the reliance on non-invasive imaging (CTA/MRA) for primary follow-up, rather than systematic DSA in all patients, may have led to an underestimation of small residual neck remnants or subtle recanalization, thereby contributing to an inflated complete occlusion rate compared to studies employing more rigorous angiographic assessment [31,32]. This represents a significant limitation when interpreting the efficacy of flow-diverting stents in this cohort. Because DSA was reserved for suspected abnormalities, the follow-up process was not blinded or uniform across treatment groups, and the sensitivity of CTA/MRA for small remnants may differ by device type, aneurysm location, and metallic artifact. The observed 98.3% complete-occlusion rate should therefore be regarded as an estimate conditioned on the imaging protocol rather than a definitive angiographic benchmark. This finding should be interpreted cautiously because follow-up imaging was not uniformly performed with DSA in all patients, and small residual neck remnants may have been underestimated on noninvasive imaging.

The WEB device is an intrasaccular flow disruptor designed primarily for the treatment of wide-necked bifurcation aneurysms. Previous studies have reported favorable angiographic outcomes after WEB treatment, with adequate occlusion rates generally in the range of approximately 83–88% [33]. The durability of WEB treatment depends on several anatomical and technical factors, particularly appropriate device sizing, aneurysm height, neck width, dome-to-neck configuration, and device shape modification [34]. Device sizing strategies, including the “+2/−1” approach, have been emphasized in practical WEB methodology to improve device apposition and reduce protrusion risk [34,35]. In the present study, early residual or recurrent filling was detected in 7 of 60 WEB-treated aneurysms, corresponding to a rate of 11.7%. This result is consistent with previous WEB literature and supports the overall angiographic durability of WEB treatment in appropriately selected aneurysms.

Aneurysm neck width has been reported as a relevant predictor of incomplete occlusion after WEB treatment [36]. Similarly, dome-to-neck configuration and other morphological parameters have been suggested to influence angiographic outcomes after intrasaccular flow disruption [37]. In our cohort, however, neck width > 4 mm was not significantly associated with early residual or recurrent filling. Mean neck width was similar between aneurysms with complete occlusion and those with early residual or recurrent filling. In contrast, aneurysm height was significantly higher in aneurysms with incomplete early occlusion. These findings suggest that, in this cohort, aneurysm height may have had a stronger association with early occlusion durability than neck width. Prior work has also suggested that the relationship between aneurysm height and WEB device height may influence incomplete occlusion after WEB treatment [38].

The WEB-treated aneurysms in the present cohort showed an adequate early occlusion profile. Previous studies have reported adequate occlusion rates after WEB treatment that are broadly comparable with our findings [39,40]. In the present series, 4 aneurysms with Raymond–Roy class 3 filling were retreated, including 2 cases treated again with the WEB device. Although neck remnants after WEB treatment may often be observed without immediate retreatment, aneurysms with persistent sac filling may require additional therapy depending on morphology, residual filling pattern, and clinical judgment. In the present study, the overall retreatment burden remained low.

The ICA subgroup analysis generated a preliminary signal that should not be interpreted as evidence of WEB inferiority. In the ICA subgroup, early residual or recurrent filling was observed in 1 of 2 coil-treated aneurysms, none of 31 flow-diverter-treated aneurysms, 2 of 14 WEB-treated aneurysms, and none of 6 aneurysms treated with stent-assisted coiling. This analysis was based on a small number of treatment-specific samples and a small number of outcome events. It was also vulnerable to confounding by indication, because the treatment modality was selected according to aneurysm morphology, bifurcation anatomy, parent-vessel anatomy, and operator judgment. The *p* value should therefore be viewed as an exploratory signal rather than as robust evidence of a treatment effect. The available aggregate data do not permit reliable adjustment for potentially relevant ICA aneurysm characteristics, such as neck width, dome-to-neck ratio, daughter sacs, branch–vessel relationships, or rupture status. Accordingly, this observation is hypothesis-generating and requires validation in larger cohorts with prespecified analyses and standardized DSA-based follow-up.

In contrast to aneurysm height, other evaluated clinical and morphological variables were not significantly associated with early residual or recurrent filling. Neck width > 4 mm did not increase the risk of incomplete early occlusion in this cohort. Similarly, patient age, sex, and hypertension were not significantly associated with early residual or recurrent filling. Previous studies have reported inconsistent results regarding the influence of sex, hypertension, and other clinical factors on recurrence after endovascular aneurysm treatment [16,17,31,41]. The absence of significant associations in the present study may be related to the limited number of outcome events. In addition, the marked non-random treatment allocation limits the extent to which observed differences can be attributed to the devices themselves.

## 5. Limitations

This study has several limitations. First, it was retrospective and based on a single-center experience, which limits generalizability. Second, only 12 early residual or recurrent filling events were observed. This event count provides inadequate statistical power for reliable multivariable modelling and makes nested subgroup analyses particularly unstable. We therefore did not interpret unadjusted *p* values as evidence of independent treatment effects, and no causal inference should be drawn from these analyses. Third, treatment selection was based on aneurysm morphology, location, bifurcation anatomy, and operator judgment, introducing potential selection bias. The treatment groups were therefore intrinsically non-equivalent: coils were preferentially used for small narrow-neck aneurysms, flow diverters for large, fusiform, or dissecting lesions, and WEB devices for wide-neck bifurcation aneurysms. The absence of a statistically significant overall difference should consequently be interpreted as a comparison within a retrospective cohort, not as proof that the modalities have comparable effectiveness. Fourth, follow-up imaging was performed using mixed modalities, including CTA, MRA, and selectively obtained DSA. DSA was not systematically performed in all patients and was generally reserved for suspected residual filling. This non-uniform ascertainment introduces verification bias and may have preferentially confirmed abnormalities detected on non-invasive imaging while missing subtle remnants in patients with apparently negative CTA/MRA examinations. The high apparent occlusion rate, particularly after flow-diverter treatment, should therefore be interpreted with caution. Fifth, the angiographic follow-up period was limited to approximately 6 months. Longer follow-up may alter occlusion rates, particularly after flow-diverting stent treatment, in which aneurysm occlusion may continue to progress over time. In summary, aneurysm height > 10 mm was associated with early residual or recurrent filling in this selected single-center cohort. No statistically significant overall difference among modalities was demonstrated, and the study was not designed or powered to establish comparative superiority. The ICA/WEB signal was based on sparse, unadjusted subgroup data and should be regarded strictly as preliminary and hypothesis-generating.

## 6. Conclusions

In this single-center cohort, aneurysm height > 10 mm was significantly associated with early residual or recurrent aneurysm filling after endovascular treatment. Overall early residual or recurrent filling rates did not differ significantly among treatment modalities. Because treatment assignment was strongly influenced by aneurysm phenotype and anatomy, this finding should not be interpreted as demonstrating equivalent performance across modalities. However, the retrospective design, small number of events, and non-uniform imaging follow-up preclude conclusions regarding comparative superiority or equivalence. The observed ICA/WEB subgroup signal is preliminary and strictly hypothesis-generating, not evidence of WEB inferiority. Larger prospective studies with prespecified analyses, balanced cohorts, adequate statistical power, and standardized DSA-based follow-up are required to evaluate comparative effectiveness.

## Figures and Tables

**Table 1:** Baseline patient and aneurysm characteristics.

Variable	Value
**Patients and demographics**	
Patients, *n*	151
Aneurysms, *n*	161
Age, years	57.22 ± 14.07
Age, median (IQR), years	58 (47–68)
Female sex	86 (56.95%)
Male sex	65 (43.04%)
Hypertension absent	68 (45.03%)
Hypertension present	83 (54.96%)
**Aneurysm morphology**	
Neck width, mm	3.04 ± 1.05
Neck width, median (IQR), mm	2.9 (2.4–3.5)
Neck-to-dome ratio	0.47 ± 0.21
Neck-to-dome ratio, median (IQR)	0.42 (0.31–0.60)
Aneurysm height, mm	7.97 ± 4.92
Aneurysm height, median (IQR), mm	7 (5–10)
Dome diameter, mm	7.71 ± 5.44
Dome diameter, median (IQR), mm	6 (4–9)
Aneurysm height ≤ 10 mm	135 (83.85%)
Aneurysm height > 10 mm	26 (16.15%)
Neck width ≤ 4 mm	130 (80.75%)
Neck width > 4 mm	31 (19.25%)
**Aneurysm location**	
ACoA	48 (29.81%)
MCA	49 (30.43%)
ICA	53 (32.92%)
Basilar artery	7 (4.35%)
Vertebral artery	4 (2.48%)
**Treatment modality**	
Coil embolization	35 (21.74%)
Flow-diverting stent	58 (36.02%)
WEB device	60 (37.27%)
Stent-assisted coiling	8 (4.97%)
**Six-month angiographic outcome**	
Complete occlusion	149 (92.55%)
Early residual or recurrent filling	12 (7.45%)

Values are presented as *n* (%), mean ± standard deviation, or median (interquartile range), as appropriate. ACoA, anterior communicating artery; ICA, internal carotid artery; MCA, middle cerebral artery; WEB, Woven EndoBridge.

**Table 2:** Six-month angiographic outcomes according to treatment modality.

Treatment Modality	Total Aneurysms, n	Complete Occlusion, n (%)	Early Residual or Recurrent Filling, n (%)	Residual/Recurrent Filling Rate (%)
Coil embolization	35	31 (88.6)	4 (11.4)	11.4
Flow-diverting stent	58	57 (98.3)	1 (1.7)	1.7
WEB device	60	53 (88.3)	7 (11.7)	11.7
Stent-assisted coiling	8	8 (100.0)	0 (0.0)	0.0
**Total**	**161**	**149 (92.5)**	**12 (7.5)**	**7.5**

Overall comparison: *p* = 0.104. Data are presented as *n* (%) unless otherwise indicated. Early residual or recurrent filling was defined as Raymond–Roy Occlusion Classification (RROC) class II or III at the 6-month angiographic follow-up. Abbreviations: RROC, Raymond–Roy Occlusion Classification; WEB, Woven EndoBridge.

**Table 3:** Factors associated with early residual or recurrent aneurysm filling.

Variable	Complete Occlusion (n = 149)	Early Residual or Recurrent Filling (n = 12)	*p* Value
**Categorical variables**			
Aneurysm height ≤ 10 mm	129 (86.6%)	6 (50.0%)	**0.005**
Aneurysm height > 10 mm	20 (13.4%)	6 (50.0%)	
Neck width ≤ 4 mm	119 (79.9%)	11 (91.7%)	0.463
Neck width > 4 mm	30 (20.1%)	1 (8.3%)	
**Continuous variables**			
Dome diameter, mm	7.61 ± 5.57	9.00 ± 3.41	0.397
Aneurysm height, mm	7.75 ± 4.85	10.67 ± 5.25	**0.048**
Neck width, mm	3.02 ± 1.06	3.25 ± 0.88	0.467
Neck-to-dome ratio	0.48 ± 0.21	0.37 ± 0.19	0.082
Age, years	57.46 ± 13.90	54.25 ± 16.34	0.448

Data are presented as n (%) or mean ± standard deviation (SD), as appropriate. Percentages for categorical variables are column percentages. Categorical variables were compared using Fisher’s exact test. Continuous variables were analyzed using Student’s *t*-test or Mann–Whitney U test, as appropriate. For categorical variables, the *p* value corresponds to the comparison across the two levels of the variable; for variables with two rows, the *p* value is shown on the first row.

## Data Availability

The datasets generated and analyzed during the current study are available from the corresponding author upon reasonable request.

## Supplementary Materials

The following supporting information can be downloaded at https://www.mdpi.com/article/10.3390/neurolint18090170/s1, Table S1: Location-specific comparison of early residual or recurrent filling according to treatment modality.

## Abbreviations

ACoA	Anterior Communicating Artery
CE-MRA	Contrast-Enhanced Magnetic Resonance Angiography
CTA	Computed Tomography Angiography
DSA	Digital Subtraction Angiography
EVT	Endovascular Treatment
ICA	Internal Carotid Artery
MCA	Middle Cerebral Artery
MRA	Magnetic Resonance Angiography
RROC	Raymond–Roy Occlusion Classification
SD	Standard Deviation
TOF-MRA	Time-of-Flight Magnetic Resonance Angiography
WEB	Woven EndoBridge
