# Supplementary material for: Early Aneurysm Occlusion Outcomes After Endovascular Treatment: A Descriptive Single-Center Cohort Evaluation
Source: Neurol Int. 2026 Sep 11;18(9):170. doi: 10.3390/neurolint18090170 (PMC13609961; doi:10.3390/neurolint18090170)
Supplement: Supplementary file 1 [file neurolint-18-00170-s001.zip › neurolint-4445264-supplementary.pdf]

Table S1. Location-specific comparison of early residual or recurrent filling according to treatment modality.

Aneurysm location	Coil embolization, residual/total (%)	Flow-diverting stent, residual/total (%)	WEB device, residual/total (%)	Stent-assisted coiling, residual/total (%)	*p* value
ACoA	1/22 (4.5)	0/6 (0)	3/20 (15.0)	0/0 (-)	0.474
MCA	1/10 (10.0)	1/17 (5.9)	2/20 (10.0)	0/2 (0)	1.000
ICA	1/2 (50.0)	0/31 (0)	2/14 (14.3)	0/6 (0)	0.019
Basilar artery	1/1 (100.0)	0/0 (-)	0/6 (0)	0/0 (-)	0.143
Vertebral artery	0/0 (-)	0/4 (0)	0/0 (-)	0/0 (-)	-

Comparisons were performed using Fisher exact test. Because of sparse cell counts, subgroup p values should be interpreted cautiously.
